# Estrogenic Effect Mechanism and Influencing Factors for Transformation Product Dimer Formed in Preservative Parabens Photolysis

**DOI:** 10.3390/toxics11020186

**Published:** 2023-02-17

**Authors:** Xiaolin Niu, Guanhui Chen, Yi Chen, Na Luo, Mei Wang, Xinyi Hu, Yanpeng Gao, Yuemeng Ji, Taicheng An

**Affiliations:** 1 Guangdong-Hong Kong-Macao Joint Laboratory for Contaminants Exposure and Health, Guangdong Key Laboratory of Environmental Catalysis and Health Risk Control, Institute of Environmental Health and Pollution Control, Guangdong University of Technology, Guangzhou 510006, China; 2 Guangzhou Key Laboratory of Environmental Catalysis and Pollution Control, Key Laboratory of City Cluster Environmental Safety and Green Development of the Ministry of Education, School of Environmental Science and Engineering, Guangdong University of Technology, Guangzhou 510006, China

**Keywords:** photolysis products, estrogenic effect mechanism, central composite design, actual water

## Abstract

The environmental transformation and health effects of endocrine disruptors (EDCs) need urgent attention, particularly the formation of transformation products with higher toxicity than parent EDCs. In this paper, an important transformation product dimer (short for ethyl 4-hydroxy-3-(2-((4-hydroxybenzoyl) oxy) ethyl) benzoate) with estrogenic activity was investigated and detected in the photolysis of preservative ethyl-paraben (EPB) dissolved in actual water. The environmental factors, such as the higher initial concentration of EPB, the stronger optical power and the lower pH could stimulate the formation of the dimer. Simultaneously, the interaction of multiple environmental factors was significant, especially the initial concentration and pH using the response surface methodology. Furthermore, the relationship between the environmental factors and the formation of the product dimer was further explained and the empirical model equation was built for predicting the amount of dimer in actual water. Quantum chemical and toxicological calculations showed the estrogenic effect mechanism of the product dimer and it was revealed further that the hydrogen bonds of the dimer and ERα proteins (ARG-394, Glu-353, His-524, GYY-521) were formed, with a lowest binding energy of −8.38 Kcal/mol during molecular docking. In addition, the health effect risk of the product dimer was higher than the parent compound in the blood, cardiovascular system, gastrointestinal system, kidney and liver. In short, the present study was of great significance for the transformation product in pollution control and health effects in the photolysis of EDCs.

## 1. Introduction

Due to the widespread occurrence of endocrine disrupting compounds (EDCs) in water, there is great attention being paid to their environmental behaviors and adverse effects on human health [1,2]. EDCs may alter the endocrine and homeostatic systems [3]. It is a risk factor in the development of tumors, such as testis, prostate, thyroid and breast cancer [4,5,6]. Once released into the environment, EDCs could undergo many transformations including photolysis, resulting in the formation of various products. Among these transformation products, some could retain the toxicity and even yield higher toxicity than parent EDCs. However, the transformation products of EDCs have received little attention [7], and the formation of toxic products under actual water has rarely been examined.

Parabens (PBs), an important group of EDCs, could bind to estrogenic receptors (ER), causing a series of adverse health effects. For instance, the exposure to PBs was related to the cancer incidence, even at negligible concentration [8,9,10]. PBs are mainly used in food additives, pharmaceuticals and personal care products as the most common typical preservative at present [2,11]. Due to the steady increase in the annual global consumption, PBs are detected ubiquitously in various environmental matrices, with concentrations up to µg/L levels in a water environment [12,13]. For instance, the maximum concentration of EPB was about 30 µg/L in surface water from the Mogi Guaçu River [14]. Even in the human body, intact PBs have been found, and are a result of long-term exposure to low doses [15,16]. Moreover, recent research shows that the potential risk is not always removed with the parent EDCs’ elimination, and some products are reported to have higher toxic properties than parents during the environmental transformation [7,17]. 

Photo-driven transformation is a simple, cost-efficient and effective approach to remove PBs quickly [18]. However, the estrogenic activity does not disappear with the elimination of the parent compound. For example, the estrogenic activity decreases by 40% during EPB degradation and then remains practically unaltered under simulated solar radiation in zinc oxide (ZnO) suspensions [19]. Moreover, the increased estrogenic activity was observed under photolysis [20]. *p*-hydroxybenzoic acid (PHBA), as the common transformation product of parabens, is detected during the photo-driven transformation of PBs in laboratory experiments and in real environmental samples [21]. The estrogenic activity of PHBA was lower than the parent compound or even no estrogenic effect [10]. However, our previous study reported that the estrogenic activity increased during the EPB photolysis, and that a new transformation product, ethyl 4-hydroxy-3-(2-((4-hydroxybenzoyl) oxy) ethyl (hereinafter, dimer), had a higher estrogen effect compared with the parent compound [20]. This finding contributed to growing concerns about the environmental and health effects of the transformation products such as this dimer. Accordingly, it raises a new question if the product dimer with higher estrogenic activity could be formed in the actual water. Furthermore, the potential influence of environmental factors on the formation of product dimer needs to be investigated. 

Computational toxicology integrates information and data from a variety of sources to develop mathematical and computer-based models to better understand and effectively predict adverse health effects caused by chemicals [22] such as EDCs. Computational toxicology assesses safety without animal testing [23], saves costs and increases efficiency. In some cases, the computer predictions could perform even better than traditional animal experiments [24,25]. Thus, the estrogenic mechanism and health effects of the product dimer were investigated via computational toxicology. In addition, the formation of the photolysis product dimer was detected using high performance liquid chromatography quadrupole time-of-flight tandem mass spectrometry (HPLC-TOF-MS) in ultra-pure water and actual water. Simultaneously, the influence of relevant environmental factors including the initial concentration, optical power and pH of the solution on the photolysis product dimer formation was assessed individually. Using the response surface methodology, the environmental factors were put together for a synthetic assessment of the product dimer formation further, a prediction model was also built. Simultaneously, the probability of health effects was predicted via the ACD/Percepta platform.

## 2. Materials and Methods

### 2.1. Chemicals

Ethyl-paraben (EPB) was supplied by Tokyo Chemical Industry, Japan (99% pure). Hydrochloric acid (HCl) and sodium hydroxide (NaOH) were purchased from Guangzhou Chemical Reagent Factory (analytically pure). Acetic acid and acetonitrile were obtained from ANPEL Laboratory Technologies (Shanghai) Inc (HPLC-grade). Ultra-pure water (18.2 MΩ cm, Millipore Corp., Burlington, MA, USA) and surface water taken from the Pearl River were used directly without other treatment. 

### 2.2. Photolysis Experiment

The photolysis of EPB was conducted in a reactor equipped with a high-pressure mercury lamp (200–500 W, maximum emission wavelength at 365 nm, Bilon, Inc., Shanghai, China) as the irradiation source, with a double-walled cooling water jacket in order to keep the constant room temperature of solutions throughout all experiments. An amount of 30 mL of each sample solution was added into a quartz test tube, which was vertically placed outside the glass well at the fixed distance. An amount of 1 mL of reaction solution was collected at required time intervals for the concentration analysis of EPB and photolysis product dimer.

### 2.3. Analytical Methods

The peak areas of EPB and its transformation product dimer were determined using a high-performance liquid chromatography quadrupole time-of-flight tandem mass spectrometry (HPLC-TOF-MS) instrument (Agilent G6545B, Santa Clara, CA, USA) in negative mode electrospray ionization (ESI^−^) with an initial fragmentor voltage of 175 V. A 5 μL injection volume was analyzed via an Agilent Ecilpse Plus C18 column (2.1 × 50 mm, 1.78 μm particle size) with a mobile phase (0.2 mL/min) consisting of acetonitrile (solvent A) and water containing 0.07% acetic acid (solvent B). The elution gradient was as follows: 0–2 min, 90% B; 2–20 min, 90% to 85% B; 20–40 min, 85% to 70% B; 40–50 min, 70% to 65% B; 50–60 min, 65% to 50% B; 60–61 min, 50% to 90% B; 61–63 min, 90% B [20]. 

### 2.4. Design of Experiment Using RSM

Response surface methodology (RSM) is a statistical method and a collection of mathematical data which can be used to model and analyze the multivariable response values [26]. The Design-Expert 12.0 software was used for the central composite experiment (CCD) design and related data analysis. The environmental factors were selected—such as a low, central and high value, namely for the initial EPB concentration (A), optical power (B) and the acidity and alkalinity of the solution (C)—as independent variables to evaluate their influence on the response value and the investigated levels were listed in Table 1. Each experiment was repeated three times and the mean value taken. A total of 18 experiments were operated in a random order to minimize systematic errors. The value of F was used to evaluate the statistical significance of the model [27].

### 2.5. Quantum Chemistry and Toxicology Calculations

The electronic structures of EPB and product dimer were optimized using Gaussian 09 software [28]. The hybrid density functional B3LYP method with the 6-31G(d,p) basis set was used, and the solvent effect in the aqueous environment was simulated using the continuum solvation model (CPCM) [29]. The estrogen receptor protein ERα (PDB ID:3UUD) removed solvent water molecules as well as excess small molecules via Pymol software (Version 2.5.2). The optimized structure and protein were further docked in Autodock 4.2 [30]. All amino acids of the protein receptor were kept rigid while the ligand molecules were flexible. The active sites of the receptor protein were covered using the docking box. The Lamarck Genetic Algorithm (LGA) was chosen as the docking engine with default parameters. The results obtained after molecular docking 60 times were analyzed visually in Pymol software (Schrödinger LLC, New York, NY, USA). Moreover, ACD/Percepta platform is an industry-leading tool of property prediction and design optimization, which was used to predict the probability of health effects on the blood, cardiovascular system, gastrointestinal system, kidney, liver and lungs.

## 3. Results and Discussion

### 3.1. Formation of Product Dimer during Photolysis

**Figure 1:**
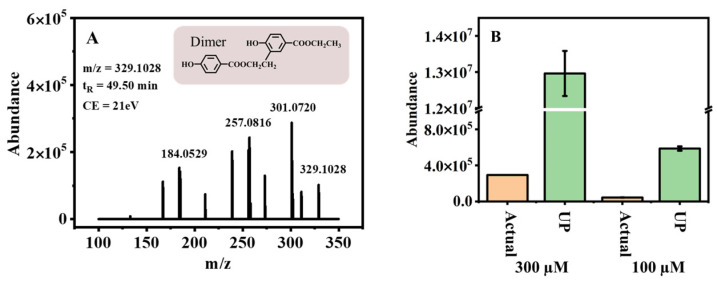
(**A**) MS/MS of transformation product dimer; (**B**) the formation in different concentration (300, 100 µM) under two different water quality conditions (actual—actual water, UP—ultra pure water).

The photochemical degradation of 300 μM EPB dissolved in ultra-pure water was performed under 500 W high-pressure mercury lamp irradiation. The extracted ion chromatogram (EICs) (Figure S1) results showed 54.7% EPB was degraded during the 120 min photolysis and some transformation products were formed, such as PHBA, 3,4-dihydroxy-benzoic acid ethyl ester (3,4-OH-EPB), and the dimer, etc. Of particular importance was the confirmation that the transformation product dimer had a higher endocrine-disrupting effect than the parent EPB [20]. As shown in Figure 1A, Table S2, the formation of the transformation product dimer at the deprotonated molecular mass of (m/z [M-H]^−^) 329.1028 was detected, and its characteristic fragment ions at m/z 301.0720, 257.0816 and 184.0529 with the retention times of 49.50 min. The MS/MS fragmentation scheme of the product dimer was shown in Figure S2. The characteristic ions and fragmentation patterns of the product dimer were discussed in our previous study [20]. Thus, the formation of such toxic transformation products in actual water environments requires urgent attention. Hence, the photochemical degradation was studied by preparing 300 µM dissolved in Pearl River water. The transformation product dimer was also detected, with the peak area 44-fold lower than in ultra-pure water after 90 min of degradation (Figure 1B). When the concentration of EPB was reduced to 100 µM, the transformation product dimer still existed, and the peak area was 14-fold lower than in ultra-pure water. The phenomenon may be explained by considering that numerous reactive radicals exist in the actual water, and they could further degrade the intermediates of the dimer. As a result, less dimer could be formed in actual water samples. Nevertheless, due to the adverse health effects of EDCs, even at extremely low concentrations, they are still of great concern. Therefore, the relationship between environmental factors and the formation of the transformation product dimer becomes an urgent issue.

### 3.2. Influence of Environmental Factors on Dimer Formation

**Figure 2:**
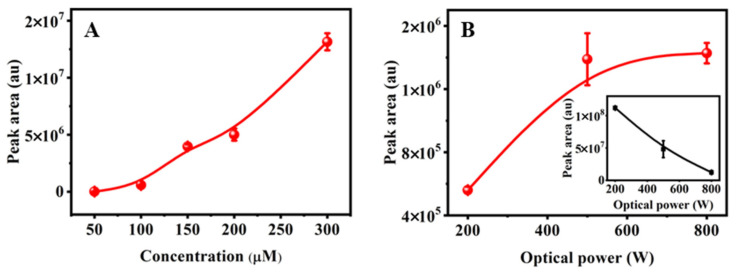
(**A**) The formation of transformation product dimer under different the initial concentration of EPB; (**B**) optical power at 90 min of photodegradation. The inset showed remaining EPB peak area.

Environmentally realistic conditions such as the concentration of the parent EPB and water quality parameters may affect the formation of the product dimer. Firstly, the influence of EPB concentration on the formation of the product dimer was investigated using different initial concentrations (300, 200, 150, 100 and 50 µM) at 90 min irradiation under 500 W. Seen from Figure 2A, it was observed that the peak area of the product dimer increased 980 times as the initial concentration increased from 50 to 300 µM. The initial concentration of EPB was positively correlated with the formation of the product dimer. That is, the higher the initial concentration, the more the formation of the product dimer. In addition, it is worth noting that the product dimer was hardly detected with the initial concentration of EPB at 50 µM. This indicates that the product dimer could not be formed when the EPB concentration was below this value (<50 µM), neglecting the influence of the water quality parameters.

Similarly, the photodegradation of initial EPB at a concentration of 160 μM was performed under different optical powers (200, 500 and 800 W) with the pH adjusted to 6 in all solutions. The peak areas of the product dimer and remaining EPB were measured as shown in Figure 2B. The remaining EPB at 200 W was about two times and eight times higher than the remaining EPB at 500 W and 800 W after 90 min degradation, respectively. These data indicate that the high optical power of the light source could promote photochemical degradation of EPB. In addition, the photodegradation peak area of EPB fit well with the pseudo-first-order kinetics equations and R^2^ was 0.98. The constant rate was obtained as 0.0037 w^−1^ (Figure S3). However, a different case was observed in the product dimer. That is, the peak area of the product dimer formed at 800 W was close to that at 500 W, and about 2.5 times higher compared to 200 W. In particular, the peak area of the product dimer had a slight increase from 500 to 800 W. There was no linear change between the peak area of the product dimer and the investigated optical power of the light source. Therefore, the influence of product dimer formation was more significant at a low optical power.

**Figure 3:**
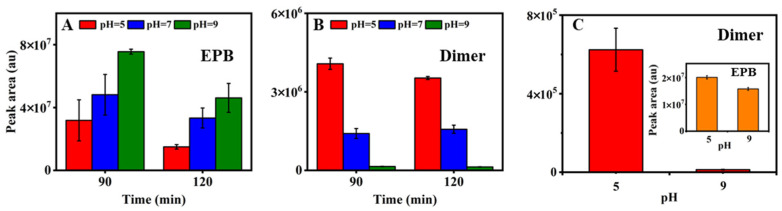
The remaining EPB peak area (**A**) and peak area of dimer (**B**) under different pH (pH = 5, 7, 9, 160 μM, 500 W). The peak area of the dimer under pH = 5, 9, 20 μM, 200 W (**C**); the inset showed peak area of remaining EPB.

Further, the influence of pH on the formation of the product dimer was investigated according to the actual water environment pH at 5, 7 and 9 (Figure 3A,B). The EPB degradation could be accumulated when the pH decreased from 9 to 5. The remaining EPB at a pH = 5 was 2.3 times lower than that at a pH = 9 after 90 mins of irradiation. When the irradiation continued to 120 min, it was observed that the remaining EPB at a pH = 5 was still lower than that of a pH = 9. Accordingly, the peak area of the product dimer formed at a pH = 5 was 2.8 times higher than a pH = 7, and 26.6 times than a pH = 9 after 90 mins of irradiation. The results revealed that, compared to the alkaline water environment, the acidic condition appears to be more favorable for EPB degradation, resulting in the formation of more product dimer. From Figure 3B, it was observed that the product dimer formation decreased with the photolysis time going from 90 to 120 min at a pH = 5 but increased at a pH = 7 and a pH = 9. At the same degradation time, the most dimer formation was observed at a pH = 5. The reason may be that the ester bonds of the dimer were relatively stable under acidic conditions. Hence, it can be concluded that the product dimer could be favorably formed under acidic conditions. The findings indicate that the product dimer could be formed under acidic conditions; even the EPB concentration is below 50 μM. In order to explore the complex effect of both the pH and EPB concentration on the formation of the product dimer, the new experiment was designed with the lower concentration of EPB at 20 μM. The result was shown in Figure 3C, and the product dimer was indeed formed. When the degradation of EPB at a pH = 5 under 30 mins of irradiation was nearly the same as that at a pH = 9 under 120 mins of irradiation, the formation of the product dimer under the acidic condition was 47.9 times higher than that under the alkaline condition. The results confirmed that an acidic condition was propitious for forming the product dimer.

### 3.3. Multiple Factors Interaction for the Formation of Product Dimer

Response surface methodology (RSM) is not only used to determine the optimum conditions of a process, but also to observe the relationship between multiple environmental variables and responses. The interaction influence of multiple factors on dimer formation was studied via a synthetic assessment based on the central composite experiment (CCD) of RSM. The results were presented in Table S1. A final empirical model equation was built to show the relationship between independent environmental factors, and the formation of product dimer was obtained successfully as follows:

$$\begin{array}{c} Y=1.434{\;\text{×}\;10}^{6}+2.105{\;\text{×}\;10}^{6}\;A+4.340{\;\text{×}\;10}^{5}\;B\;-\;3.278{\;\text{×}\;10}^{6}\;C+2.087{\;\text{×}\;10}^{6}\;\mathrm{AB}\;-\;3.159{\;\text{×}\;10}^{6}\;\mathrm{AC}\;-\;1.797{\;\text{×}\;10}^{6}\;\mathrm{BC}+6.443{\;\text{×}\;10}^{5}\;A^{2}\;\\ \;-\;4.673{\;\text{×}\;10}^{5}\;B^{2}+1.949{\;\text{×}\;10}^{6}\;C^{2}\;-\;1.932{\;\text{×}\;10}^{6}\;\mathrm{ABC}+1.512{\;\text{×}\;10}^{6}{\;A}^{2}B\;-51,801.39{\;A}^{2}C+1{.271\;\text{×}\;10}^{6}{\;\mathrm{AB}}^{2} \end{array}$$

where Y was the peak area of the product dimer, and A, B and C correspond to the initial concentration, optical power and pH, respectively.

The analysis of variance (ANOVA) was used to evaluate the significance and reliability of the prediction model, and the results were depicted in Table 2. The F-value represented the ratio of the mean square and residual error of the model, and the statistical significance of the F distribution was used to estimate the *p*-value. The F value of the model was 1657.14 and the *p*-value was less than 0.05, indicating that the model was extremely significant (*p* < 0.0001). Meanwhile, the *p*-value of the lack of fit (0.6168) implied that the pure error was not significant (>0.05), thus confirming the adequacy of the model. Additionally, the small difference between coefficient of determination R^2^ and the corresponding adjusted R^2^ (0.9998 and 0.9992, respectively) indicated that unnecessary factors were not included in the model. Then, the legitimacy of the model was further validated due to a high correlation between the experimental and predicted values of the model (Figure S4). To sum up, the model was accurate and applicable to describe the formation of the product dimer.

**Figure 4:**
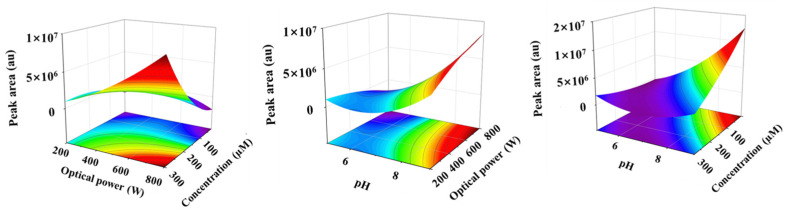
Interactive influence of two factors on the formation of product dimer.

In the empirical model, the positive and negative influences on the response value were represented by the sign (+) and sign (−) before the coefficients, respectively. The model term was significant, with a smaller probability value (*p* < 0.05). According to the ANOVA analysis in Table 2, three relevant environmental factors were all significant, with a *p*-value less than 0.0001. Compared to optical power, the variables of the initial EPB concentration and pH of the solution had relatively more significance, which was consistent with the analysis of the single factor. In addition, the interaction of the response value between the corresponding variables was significant (*p* < 0.0001). Moreover, the three-dimensional (3D) response surface and the corresponding two-dimensional (2D) contour plots were drawn by keeping one variable fixed at a central level of zero (Figure 4). The red vertex was clearly obtained by measuring the combined effects of the two environmental factors, that is, both the higher initial EPB concentration and the stronger optical power will be beneficial to the formation of the product dimer when the pH of the solution was at 7. Similarly, the higher optical power and the strong acidic conditions produced the same result under the initial concentration of EPB at 160 µM. Obviously, more product dimer was formed in both the stronger acidic and the higher initial EPB concentration conditions under 500 W than the above two conditions. The simultaneous interaction would be easier to form the product dimer. For example, the product dimer was undetected under 500 W and with the pH at 7; however, it was found under 200 W and with the pH at 5 with the initial EPB concentration of 20 µM. Under the irradiation of 500 W, the peak area of the product dimer was unextractable in the degradation of the 50 µM EPB concentration. However, the formation of the product dimer appeared during photodegradation when the initial EPB concentration was reduced to 20 µM, with the pH of the solution at 5. Furthermore, the simultaneous interaction of the relevant environmental factors was also significant, with the *p*-value less than 0.0001. In short, the formation of the product dimer could be accelerated by the complex interaction of all the three environmental factor initial EPB concentrations, optical power and pH in water environment.

In an actual water environment, the parent compound EPB was continuously discharged into the aqueous environment; the product dimer may be formed under solar irradiation, especially in summer when the light intensity was relatively strong. The formation of the product dimer was easier in weakly acidic, actual water. Hence, the formation of the product dimer should be regarded and prevented in these environmental waters with severe pollution, higher light intensity and acidic water.

### 3.4. Health Effect of Product Dimer

**Figure 5:**
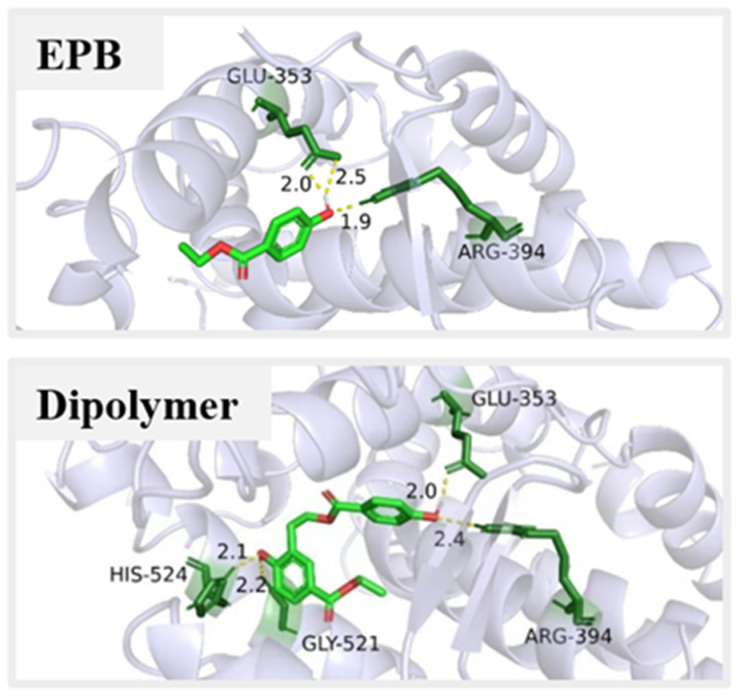
The docking result of EPB and product dimer with estrogen receptor α (ERα).

The product dimer with adverse impacts on environment and human health should also be seriously studied. For example, PBs may play some role via the estrogen-related receptor γ in the carcinogenesis of human breast cancer, due to hydrogen bonds forming between the *p*-hydroxyl group of PBs and the Glu275/Arg316 of the oestrogen-related receptor γ [31]. Our early research showed that the formation of the product dimer was responsible for the increased estrogenic activity during the photolysis of EPB. It was quite reasonable to suppose that the product dimer had a higher estrogenic activity than the original parent EPB [20]. Further, the mechanisms of the estrogenic effects of the product dimer were investigated using the theoretical calculation. Figure 5 showed the interaction pattern of EPB and the photolysis product dimer binding to ERα. The hydrogen bonds were formed between the hydroxyl group of EPB and the Arg 346 and GLU 353 amino group of ERα. Similarly, the two hydrogen bonds were also obtained for the product dimer. In addition, the new hydrogen bonds were also formed between another hydroxyl group of the product dimer and the HIS-524 and GLY-521 amino group of ERα. The lowest binding energy of the product dimer (−8.38 Kcal/mol) with ERα proteins was lower than EPB (−5.27 Kcal/mol). These data suggest that the product dimer had a higher affinity than the parent EPB, resulting in the formation of a more stable complex with ERα proteins. That is, the product dimer had higher endocrine disrupting effects than parent EPB.

Besides, the probability of health effects on the blood, cardiovascular system, gastrointestinal system, kidney, liver, lungs was predicted using the ACD/Percepta platform. The probability (*p*) value between zero and one was predicted and the results were shown in Table 3. The higher the *p* value, the greater the probability of health risks. Compared to the parent EPB, the product dimer has a higher impact on the test tissues and organs of human beings, except on human lungs. In particular, the most serious effect was observed on the cardiovascular system of human health, and the atomic/functional group contributions to the calculated parameter values are highlighted in Figures S5 and S6. In short, the health effects of the product dimer require urgent attention.

## 4. Conclusions

Several transformation products of EDCs could have higher toxicity than the parent compounds, and therefore pose a great threat to human health. In this study, an important transformation product dimer was investigated during the EPB photodegradation, and it was detected not only in ultra-pure water but also in actual water. The formation of the product dimer was closely related to the environmental factors, the initial EPB concentration, optical power and pH of the solution. The higher the initial concentration and optical power, the more the product dimer could be formed. In addition, the acidic environment could be favorable for the formation of the product dimer. What is more, the interaction of multiple factors had a significant influence on the formation of the product dimer, particularly the combination of the initial EPB concentration and the pH of the solution. The empirical model equation showing the relationship between the environmental factors and the formation of the product dimer was built. Additionally, the estrogenic effect mechanism was further revealed and the results showed that compared to the parent EPB, more hydrogen bonds were formed between the product dimer and ERα proteins (ARG-394, Glu-353, His-524, GYY-521) and the binding energy was lower (dimer: −8.38 < EPB: −5.27 Kcal/mol). The health effect risks of the product dimer were higher than the parent EPB in human blood, the kidney, liver, cardiovascular and gastrointestinal systems. The most serious effect was observed on the cardiovascular system of human beings. Despite recent research increasingly focusing on the parent EDCs, gaps in the knowledge surrounding the transformation products and their adverse effects remain. The formation and adverse effects of toxic products especially should be paid more attention in future studies and risk assessments.

## Figures and Tables

**Table 1:** Experimental factors and their levels.

Factors	Symbols	Levels		
		−1	0	1
Initial concentration (µM)	A	20	160	300
Optical power (W)	B	200	500	800
pH	C	5	7	9

**Table 2:** ANOVA for the multiple equation.

Source	Sum of Squares	df	Mean Square	F-Value	*p*-Value
Model	4.31 × 10^14^	13	3.32 × 10^13^	1657.14	<0.0001
A-C0	8.86 × 10^12^	1	8.86 × 10^12^	442.92	<0.0001
B-P	3.77 × 10^11^	1	3.77 × 10^11^	18.83	0.0123
C-pH	2.15 × 10^13^	1	2.15 × 10^13^	1073.71	<0.0001
AB	3.48 × 10^13^	1	3.48 × 10^13^	1740.63	<0.0001
AC	7.98 × 10^13^	1	7.98 × 10^13^	3988.78	<0.0001
BC	2.58 × 10^13^	1	2.58 × 10^13^	1290.94	<0.0001
A^2^	1.13 × 10^12^	1	1.13 × 10^12^	56.2	0.0017
B^2^	5.92 × 10^11^	1	5.92 × 10^11^	29.57	0.0056
C^2^	1.03 × 10^13^	1	1.03 × 10^13^	514.13	<0.0001
ABC	2.99 × 10^13^	1	2.99 × 10^13^	1491.7	<0.0001
A^2^B	3.66 × 10^12^	1	3.66 × 10^12^	182.65	0.0002
A^2^C	4.29 × 10^9^	1	4.29 × 10^9^	0.2145	0.6673
AB^2^	2.58 × 10^12^	1	2.58 × 10^12^	129.09	0.0003
Residual	8.01 × 10^10^	4	2.00 × 10^10^		
Lack of Fit	7.49 × 10^9^	1	7.49 × 10^9^	0.3095	0.6168
Pure Error	7.26 × 10^10^	3	2.42 × 10^10^		
Cor Total	4.31 × 10^14^	17			
Std.Dev.	1.415 × 10^5^		R^2^		0.9998
Mean	2.615 × 10^6^		Adj R^2^		0.9992
C.V.%	5.41		Pred R^2^		0.9753
PRESS	1.06 × 10^13^		Adep Precision		170.0202

**Table 3:** Probability (*p*) value of health effects.

	Blood	Cardiovascular System	Gastrointestinal System	Kidney	Liver	Lungs
Parent EPB	0.07	0.15	0.22	0.05	0.04	0.22
Product Dimer	0.14	0.34	0.28	0.25	0.1	0.19

## Data Availability

The authors confirm that the data supporting the findings of this study are available with the corresponding author.

## Supplementary Materials

The following supporting information can be downloaded at: https://www.mdpi.com/article/10.3390/toxics11020186/s1, Figure S1: The extracted ion chromatograms (EICs) chromatograms of the 300 μM EPB photochemical degradation; Figure S2: The MS/MS fragmentation scheme of product dimer; Figure S3: A plot of pseudo-first order rate constant vs. optical power. Figure S4: Predicted versus actual values plot for the formation of product dimer; Figures S5 and S6: The atomic/functional group contributions to the calculated parameter values are highlighted on EPB and product dimer; Table S1: Experimental design matrix and values of response. Table S2: The [M-H]^−^, retention times and mass error of product dimer.

## References

[1] Mboula V.M., Hequet V., Andres Y., Gru Y., Colin R., Dona-Rodriguez J., Pastrana-Martinez L., Silva A., Leleu M., Tindall A. (2015). Photocatalytic degradation of estradiol under simulated solar light and assessment of estrogenic activity. Appl. Catal. B-Environ..

[2] Lincho J., Martins R.C., Gomes J. (2021). Paraben Compounds-Part I: An Overview of Their Characteristics, Detection, and Impacts. Appl. Sci..

[3] Amir S., Shah S.T.A., Mamoulakis C., Docea A.O., Kalantzi O.I., Zachariou A., Calina D., Carvalho F., Sofikitis N., Makrigiannakis A. (2021). Endocrine Disruptors Acting on Estrogen and Androgen Pathways Cause Reproductive Disorders through Multiple Mechanisms: A Review. Int. J. Environ. Res. Public Health.

[4] Kahn L.G., Philippat C., Nakayama S.F., Slama R., Trasande L. (2020). Endocrine-disrupting chemicals: Implications for human health. Lancet Diabetes Endocrinol..

[5] Adegoke E.O., Rahman M.S., Park Y.J., Kim Y.J., Pang M.G. (2021). Endocrine-Disrupting Chemicals and Infectious Diseases: From Endocrine Disruption to Immunosuppression. Int. J. Mol. Sci..

[6] Zoeller R.T., Brown T.R., Doan L.L., Gore A.C., Skakkebaek N.E., Soto A.M., Woodruff T.J., Saal F.S.V. (2012). Endocrine-Disrupting Chemicals and Public Health Protection: A Statement of Principles from The Endocrine Society. Endocrinology.

[7] Escher B.I., Stapleton H.M., Schymanski E.L. (2020). Tracking complex mixtures of chemicals in our changing environment. Science.

[8] Darbre P.D., Harvey P.W. (2008). Paraben esters: Review of recent studies of endocrine toxicity, absorption, esterase and human exposure, and discussion of potential human health risks. J. Appl. Toxicol..

[9] Darbre P.D., Aljarrah A., Miller W.R., Coldham N.G., Sauer M.J., Pope G.S. (2004). Concentrations of parabens in human breast tumours. J. Appl. Toxicol..

[10] Routledge E.J., Parker J., Odum J., Ashby J., Sumpter J.P. (1998). Some alkyl hydroxy benzoate preservatives (parabens) are estrogenic. Toxicol. Appl. Pharm..

[11] Haman C., Dauchy X., Rosin C., Munoz J.F. (2015). Occurrence, fate and behavior of parabens in aquatic environments: A review. Water Res..

[12] Styszko K., Proctor K., Castrignano E., Kasprzyk-Hordern B. (2021). Occurrence of pharmaceutical residues, personal care products, lifestyle chemicals, illicit drugs and metabolites in wastewater and receiving surface waters of Krakow agglomeration in South Poland. Sci. Total Environ..

[13] Taylor K.W., Troester M.A., Herring A.H., Engel L.S., Nichols H.B., Sandler D.P., Baird D.D. (2018). Associations between Personal Care Product Use Patterns and Breast Cancer Risk among White and Black Women in the Sister Study. Environ. Health Perspect..

[14] Galinaro C.A., Pereira F.M., Vieira E.M. (2015). Determination of Parabens in Surface Water from Mogi Guau River (So Paulo, Brazil) Using Dispersive Liquid-Liquid Microextraction Based on Low Density Solvent and LC-DAD. J. Braz. Chem. Soc..

[15] Wang L., Kannan K. (2013). Alkyl protocatechuates as novel urinary biomarkers of exposure to p-hydroxybenzoic acid esters (parabens). Environ. Int..

[16] Bledzka D., Gromadzinska J., Wasowicz W. (2014). Parabens. From environmental studies to human health. Environ. Int..

[17] Escher B.I., Fenner K. (2011). Recent Advances in Environmental Risk Assessment of Transformation Products. Environ. Sci. Technol..

[18] An T.C., Fang H.S., Li G.Y., Wang S.L., Yao S.D. (2014). Experimental and Theoretical Insights into Photochemical Transformation Kinetics and Mechanisms of Aqueous Propylparaben and Risk Assessment of Its Degradation Products. Environ. Toxicol. Chem..

[19] Frontistis Z., Antonopoulou M., Venieri D., Dailianis S., Konstantinou I., Mantzavinos D. (2017). Solar photocatalytic decomposition of ethyl paraben in zinc oxide suspensions. Catal Today.

[20] Gao Y.P., Niu X.L., Qin Y.X., Guo T., Ji Y.M., Li G.Y., An T.C. (2020). Unexpected culprit of increased estrogenic effects: Oligomers in the photodegradation of preservative ethylparaben in water. Water Res..

[21] Petala A., Frontistis Z., Antonopoulou M., Konstantinou I., Kondarides D.I., Mantzavinos D. (2015). Kinetics of ethyl paraben degradation by simulated solar radiation in the presence of N-doped TiO2 catalysts. Water Res..

[22] Reisfeld B., Mayeno A.N. (2012). What is computational toxicology?. Methods Mol. Biol..

[23] Daston G.P., Mahony C., Thomas R.S., Vinken M. (2022). Assessing Safety Without Animal Testing: The Road Ahead. Toxicol. Sci..

[24] Mostrag-Szlichtyng A., Comenges J.M.Z., Worth A.P. (2010). Computational toxicology at the European Commission’s Joint Research Centre. Expert Opin. Drug Metab. Toxicol..

[25] Knudsen T.B., Spencer R.M., Pierro J.D., Baker N.C. (2020). Computational biology and in silico toxicodynamics. Curr. Opin. Toxicol..

[26] Bezerra M.A., Santelli R.E., Oliveira E.P., Villar L.S., Escaleira L.A. (2008). Response surface methodology (RSM) as a tool for optimization in analytical chemistry. Talanta.

[27] Liu Z.H., Lan H., Wang Y., Zhang J.S., Qin J., Zhang R.X., Dong N. (2022). Highly efficient degradation of bisphenol A with persulfate activated by vacuum-ultraviolet/ultraviolet light (VUV/UV): Experiments and theoretical calculations. Chem. Eng. J..

[28] Frisch M.J., Trucks G.W., Schlegel H.B., Scuseria G.E., Robb M.A., Cheeseman J.R., Scalmani G., Barone V., Mennucci B., Petersson G.A. (2009). Gaussian 09.

[29] Gao Y.P., Ji Y.M., Li G.Y., An T.C. (2016). Theoretical investigation on the kinetics and mechanisms of hydroxyl radical-induced transformation of parabens and its consequences for toxicity: Influence of alkyl-chain length. Water Res..

[30] Lu J.J., Zhou F.M., Hu X.J., Fang J.J., Liu C.X., Zhu B.Q., Ding Z.S. (2020). Molecular docking simulation and in vitro studies on estrogenic activities of flavonoids from leaves of Carya cathayensis Sarg. Steroids.

[31] Zhang Z.B., Sun L.B., Hu Y., Jiao J., Hu J.Y. (2013). Inverse antagonist activities of parabens on human oestrogen-related receptor gamma (ERR gamma): In vitro and in silico studies. Toxicol. Appl. Pharmacol..

