# Supplementary material for: Estrogenic Effect Mechanism and Influencing Factors for Transformation Product Dimer Formed in Preservative Parabens Photolysis
Source: Toxics. 2023 Feb 17;11(2):186. doi: 10.3390/toxics11020186 (PMC9959869; doi:10.3390/toxics11020186)
Supplement: Supplementary file 1 [file toxics-11-00186-s001.zip › toxics-2167515-supplementary.pdf]

Estrogenic Effect Mechanism and Influencing Factors for Transformation Product Dimer Formed in Preservative Parabens Photolysis

Xiaolin Niu [1,2], Guanhui Chen [1,2], Yi Chen [1,2], Na Luo [1,2], Mei Wang [1,2], Xinyi Hu [1,2], Yanpeng Gao [1,2,*], Yuemeng Ji [1,2] and Taicheng An [1,2]

[1] Guangdong-Hong Kong-Macao Joint Laboratory for Contaminants Exposure and Health, Guangdong Key Laboratory of Environmental Catalysis and Health Risk Control, Institute of Environmental Health and Pollution control, Guangdong University of Technology, Guangzhou 510006, China

[2] Guangzhou Key Laboratory of Environmental Catalysis and Pollution Control, Key Laboratory of City Cluster Environmental Safety and Green development of the Ministry of Education, School of Environmental Science and Engineering, Guangdong University of Technology, Guangzhou 510006, China

* Correspondence: gaoyp2016@gdut.edu.cn

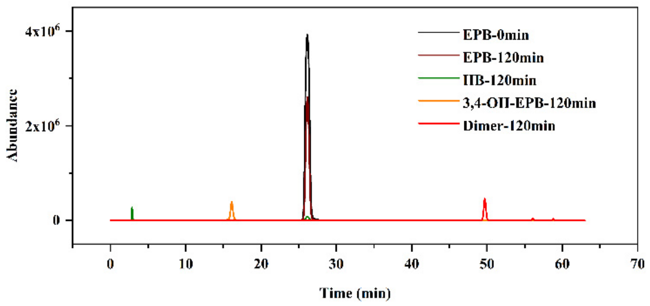

Figure S1. The extracted ion chromatograms (EICs) chromatograms of the 300 μM EPB photochemical degradation under 500W high-pressure mercury lamp irradiation.

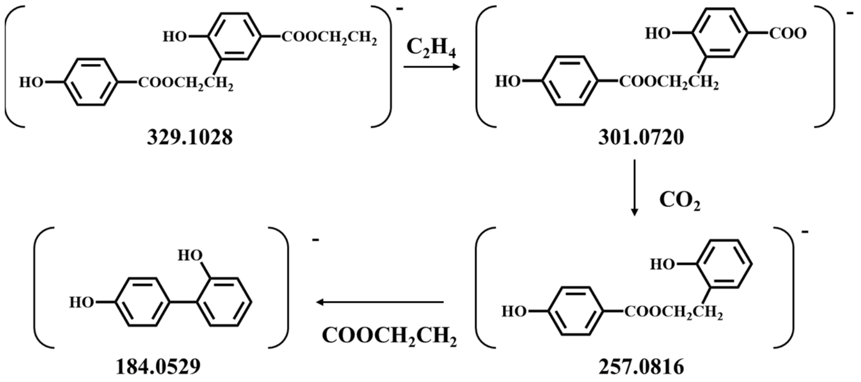

Figure S2. The MS/MS fragmentation scheme of product dimer.

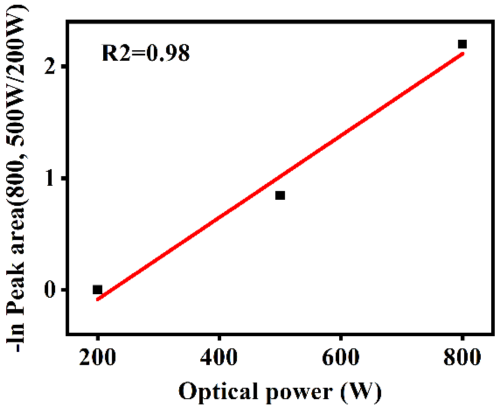

Figure S3. A plot of pseudo-first order rate constant vs. optical power.

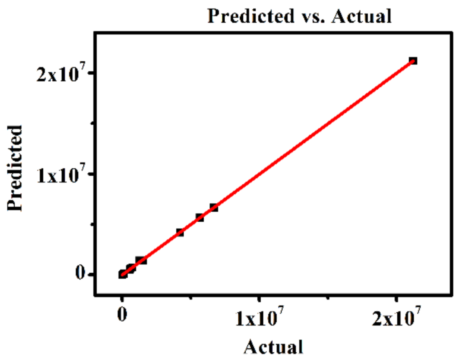

Figure S4. Predicted versus actual values plot for the formation of product dimer

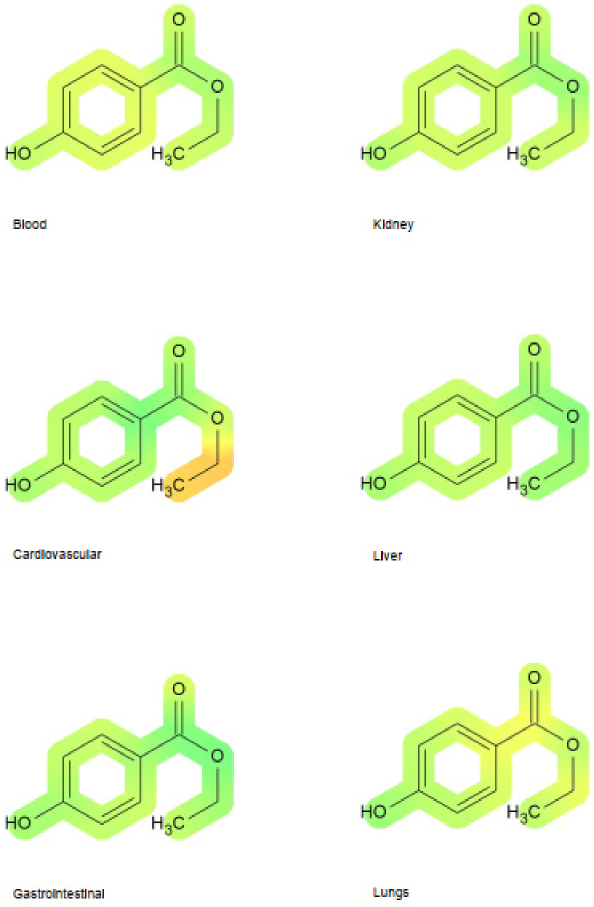

Figure S5. The atomic/functional group contributions to the calculated parameter values are highlighted on EPB

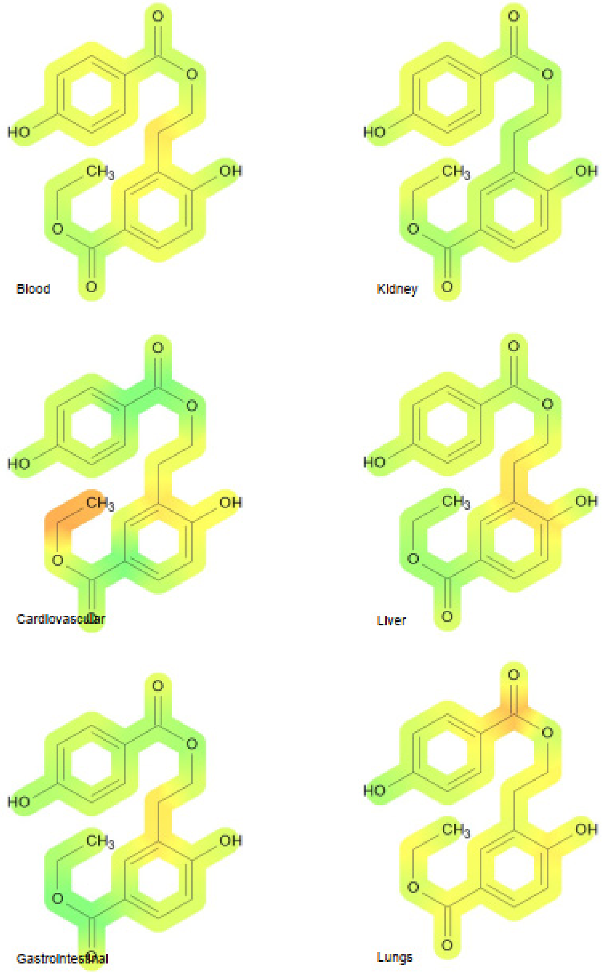

Figure S6. The atomic/functional group contributions to the calculated parameter values are highlighted on product dimer

Table S1. Experimental design matrix and values of response

Run number	Symbols			Response value	
	A	B	C	Actual values	Predicted values
1	0	0	1	131846	131800
2	0	-1	0	559681	559700
3	0	1	0	1427760	1428000
4	-1	-1	1	13020	13020
5	1	1	-1	21179027	21180000
6	1	-1	-1	5656710	5657000
7	1	-1	1	137301	137300
8	0	0	0	1272934	1273000
9	-1	1	-1	72145	72145
10	1	1	1	744156	744200
11	0	0	-1	6687576	6688000
12	-1	1	1	0	0
13	-1	0	0	0	0
14	0	0	0	1272934	1273000
15	0	0	0	1542319	1542000
16	0	0	0	1542319	1542000
17	-1	-1	-1	623926	623900
18	1	0	0	4210574	4211000

Table S2. The $[M-H]^-$, retention times and mass error of product dimer

Product	$[M-H]^-$	Retention times (min)	Mass Error (ppm)
Dimer	329.1028	49.50	0.9
